# A case of Gerstmann-Straussler-Scheinker (GSS) disease with supranuclear gaze palsy

**DOI:** 10.1186/s40734-019-0082-1

**Published:** 2019-12-11

**Authors:** Nicole A. Ufkes, Craig Woodard, Marian L. Dale

**Affiliations:** 1 College of Medicine, Medical University of South Carolina, 96 Jonathan Lucas Street, Charleston, SC 29425 USA; 2 Neurology Department, Ralph H. Johnson VA Medical Center, Charleston, SC USA; 3 Department of Neurology, Medical University of South Carolina, Charleston, SC USA; 4 Department of Neurology, Oregon Health and Science University, 3181 SW Sam Jackson Park Road, L226, Portland, OR 97239 USA

**Keywords:** Supranuclear palsy, Gerstman-Straussler-Scheinker disease, Prion disease, Gait disorders/ataxia

## Abstract

**Background:**

Gerstmann-Straussler-Scheinker disease (GSS), an autosomal dominant prion disorder, usually presents as a slowly progressive cerebellar ataxia followed by later cognitive decline. We present a member of the GSS Indiana Kindred with supranuclear palsy, a less common feature in GSS.

**Case presentation:**

A 42-year-old man presented with 12 months of progressive gait and balance difficulty. Exam was notable for ataxia and cerebellar eye movement abnormalities. Genetic testing revealed a F198S variant in the prion protein (PRNP) gene, the pathological variant of GSS associated with his family, the Indiana kindred. Eighteen months after initial presentation supranuclear palsy developed.

**Conclusions:**

GSS is a neurodegenerative prion disease with diverse clinical presentations, and exhibits greater variability in disease phenotype compared to other inherited spongiform encephalopathies. GSS should be on the differential for patients with ataxia and supranuclear palsy, and it is important to assess both horizontal and vertical saccades and optokinetic nystagmus in patients with ataxia.

## Background

Gerstmann-Straussler-Scheinker disease (GSS), an autosomal dominant prion disorder, usually presents as a slowly progressive cerebellar ataxia followed by later cognitive decline. However, GSS demonstrates a greater degree of variability in disease phenotype than in other inherited spongiform encephalopathies, with heterogeneity within and across kindreds [1, 2]. Unlike most forms of GSS, in a particular cluster of families in Indiana, the “Indiana Kindred,” abnormal eye movements are a common feature. The eye movement abnormalities in the Indiana Kindred are most often cerebellar-type abnormalities such as gaze-evoked nystagmus, rebound nystagmus, impaired smooth pursuit, and hypometric saccades. We present a case of a male member of the Indiana Kindred with supranuclear palsy, an uncommon feature in GSS. We include representative video of the finding of supranuclear palsy in this variant of GSS, a rare disease with a prevalence estimated at 1–10 per 100 million [1]. We discuss GSS as an important consideration in the differential diagnosis of supranuclear palsy.

## Case report

A 42-year-old systems administrator presented with 12 months of progressive gait and balance difficulty, head titubation, and slurred speech. His first symptom was stumbling down the stairs on dog walks. He complained “I walk like an alcoholic.” Past medical history was significant for resolved alcohol abuse, hypertension, gastroesophageal reflux disease, and bariatric surgery. Physical exam showed impaired smooth pursuit, square wave jerks in primary gaze, dysmetria, length dependent small fiber neuropathy, and ataxic gait. Horizontal and vertical saccade latencies were normal at that time. An initial brain MRI and labs for treatable causes of ataxia, including vitamin deficiencies, were both unremarkable. The patient was not in contact with his extended family, but was aware of a paternal history of neurodegenerative disease.

Genetic testing revealed a F198S variant in the PRNP gene, the pathological variant of GSS associated with his family, the Indiana kindred [1]. Eighteen months after initial presentation, in addition to worsening appendicular cerebellar signs (Additional file 1: Video S1), supranuclear palsy had developed, with limited excursion of vertical eye movements (seen on voluntary excursion and pursuit examination in Additional file 2: Video S2). The patient displayed compensatory neck extension and flexion for vertical eye movements with a frontalis sign, consistent with supranuclear palsy. Reflexive saccade speed is not shown on the video. The vestibulo-ocular reflex remained intact horizontally and vertically (not shown in video). Vestibulo-ocular reflex (VOR) cancellation and optokinetic nystagmus (OKN) were not assessed.


**Additional file 1:** **Video S1.** Cerebellar signs and dysarthria in a GSS patient



**Additional file 2: VideoS2.** Impaired smooth pursuit and vertical supranuclear palsy with reflexive neck extension, neck flexion, and frontalis sign in a GSS patient


## Discussion

This case of GSS in the Indiana Kindred highlights the development of supranuclear palsy superimposed on ataxia in GSS. The finding of supranuclear palsy in the Indiana Kindred is likely due to the different distribution of underlying pathology compared to other cases of GSS. At least 16 different prion protein gene mutations have been identified in GSS and approximately 56 kindreds are scattered throughout the world [2]. In general, GSS is characterized by pathologic PrP amyloid deposition with accompanying spongiform change occurring throughout the cortex, cerebellum, brainstem, and subcortical nuclei. In the Indiana Kindred, a point mutation in in codon 198 of the prion protein (PRNP) gene results in amyloid deposits predominantly located in the inner cortical layers [3]. In addition to amyloid deposition, this kindred uniquely features intraneuronal deposits of hyperphosphorylated tau and neurofibrillary tangles in the cortex and subcortical nuclei [1, 4].

Affected members in the GSS Indiana Kindred typically present between age 40 to 60 years, with ataxia and gait disturbance, dysarthria, oculomotor abnormalities, cognitive decline, and progressive clumsiness. Akinetic rigidity, bradykinesia, and dementia are observed in the end-stages of disease. Patients usually live after initial presentation for an average 5 years, with a range from 2 to 12 years [1].

In a study examining eye movements abnormalities in the Indiana Kindred, all patients featured abnormal eye movements of the cerebellar variety, including gaze-evoked nystagmus, rebound nystagmus, impaired smooth pursuit, and hypometric saccades [5]. Each affected member had impaired smooth pursuit and abnormal OKN, and could not suppress the VOR normally with fixation. In later disease stages, features typical of more advanced midbrain and basal ganglia disease were observed, such as supranuclear gaze palsy, decreased blinking, and a lid retraction stare. Of note, two asymptomatic kindred members in the study displayed abnormal eye movements, which the authors believed to be the earliest sign of otherwise undetectable GSS disease. Had we looked for it earlier in his disease course, it is likely that our patient would have demonstrated impairment of the downward fast phase of the OKN, which may have provided a clue to the etiology of his ataxia.

Supranuclear gaze palsy is defined as the impairment of horizontal gaze, vertical gaze, or both due to dysfunction in brainstem gaze center connections responsible for generating voluntary gaze. Vertical supranuclear palsy is attributed to pathology along the rostral interstitial nucleus of the medial longitudinal fasciculus, above the level of the ocular motor nuclei that control vertical eye movements (hence, supranuclear). Supranuclear gaze palsy, especially when affecting downward gaze, is a classical clinical feature of progressive supranuclear palsy (PSP).

The classic clinical PSP presentation, Richardson’s Syndrome, is characterized by vertical supranuclear gaze palsy and the early onset of postural instability and falls [6]. However, several other presentations of PSP are recognized, and, furthermore, supranuclear gaze palsy is not always observed in PSP patients. As a result, the 2017 Movement Disorder Society PSP diagnostic criteria address the variety of PSP clinical presentations by describing different PSP phenotypes. The criteria define 4 core clinical features: ocular motor dysfunction, postural instability, akinesia, and cognitive dysfunction. Our patient most resembled the PSP with predominant ocular motor dysfunction (PSP-OM) subtype of PSP, though certainly the ultimate prominent appendicular ataxia and family history of GSS were exclusionary for PSP.

Supranuclear palsy is also not specific for PSP diagnosis, and occurs in several other neurodegenerative disorders, including other prion diseases. For example, there are reports of familial Creutzfeldt-Jakob disease (CJD) with mutations at prion proteins codons 129 and 200 manifesting with a PSP-like phenotype, while a thalamocortical MM2 subtype can mimic PSP in sporadic CJD cases [7]. In a study analyzing autopsy data of 27 patients with supranuclear gaze palsy and parkinsonism, pathology was consistent with a variety of neurodegenerative diseases in addition to CJD, including PSP, Parkinson disease, corticobasal degeneration, and multiple system atrophy [8]. Patients with ataxia with oculomotor apraxia (AOA) and ataxia-telangectasia can also exhibit supranuclear palsy, though AOA is characterized by more severe impaired pursuit.

## Conclusion

Pronounced and early abnormalities in eye movements, such as a progressive supranuclear palsy, are a unique feature related to subcortical tau deposition in the GSS Indiana kindred [4, 5]. GSS is a neurodegenerative prion disease with diverse clinical presentations. Differing symptoms and time courses can be observed not only between different kindreds, but amongst members within the same kindred. GSS should be on the differential for patients with ataxia and supranuclear palsy, and it is important to assess both horizontal and vertical saccades and optokinetic nystagmus in patients with ataxia.

## Data Availability

Data sharing is not applicable to this article as no datasets were generated or analyzed during the current study.

## Abbreviations

AOA: Ataxia with oculomotor apraxia
CJD: Creutzfeldt-Jakob disease
GSS: Gerstmann-Straussler-Scheinker disease
OKN: Optokinetic nystagmus
PRNP: Prion protein
PSP: Progressive supranuclear palsy
PSP-OM: PSP with predominant ocular motor dysfunction
SGP: Supranuclear gaze palsy
VOR: Vestibulo-ocular reflex
